# Strain-Rate Dependence of Tensile Behavior in Commercial-Grade Tungsten—Effect of Recrystallization Condition

**DOI:** 10.3390/ma15175836

**Published:** 2022-08-24

**Authors:** Byeong Seo Kong, Ji Ho Shin, Taejeong An, Changheui Jang, Hyoung Chan Kim

**Affiliations:** 1 Department of Nuclear and Quantum Engineering, Korea Advanced Institute of Science and Technology, 291 Daehak-ro, Yuseong-gu, Daejeon 34141, Korea or; 2 Central Research Institute, Korea Hydro and Nuclear Power Co., Ltd., Daejeon 34101, Korea; 3 Korea Institute of Fusion Energy, 169-148 Gwahak-ro, Daejeon 34133, Korea

**Keywords:** tungsten, strain rate, recrystallization, tensile deformation, dynamic recrystallization

## Abstract

The tensile deformation behavior of double-forged (DF-W) and recrystallized (RX-W) commercial-grade tungsten was investigated at 700 °C. With increasing strain rate, the dominant dynamic recrystallization (DRX) mechanism changes from continuous dynamic recrystallization (CDRX) to discontinuous dynamic recrystallization (DDRX). For DF-W, pre-existing sub-grains promote CDRX and associated a high-DRX fraction, resulting in reduced post-necking strain under a static condition. With increasing strain rate, a shift in the restoration mechanism from CDRX to DDRX contributes to the enhanced ductility in DF-W, while RX-W shows enhanced flow hardening without a loss of ductility. These results suggest that the strain-rate dependence of mechanical behavior depends on the initial microstructure.

## 1. Introduction

Tungsten (W), a refractory material, has been recognized as a promising material under the extremely harsh environment of a fusion reactor, because of its high melting point, good thermal conductivity, low retention of tritium, and sputtering yield [1,2,3]. Because of its excellent properties at high temperatures, W has been adopted as a divertor target, composed of W mono-blocks with a Cu cooling tube in ITER (international thermonuclear experimental reactor) [4]. However, W has intrinsic drawbacks, such as low fracture resistance as well as high ductile-to-brittle temperature (DBTT), related to poor mobility of non-planar screw dislocations, with a very high Peierls stress during deformation [1,4,5,6,7,8,9]. In order to widen the operating temperature window, experimental studies have attempted thermo-mechanical processing (TMP) to improve the ductility or lower the DBTT of W by producing high-density edge dislocations, contrary to classic work hardening behavior [1,5,6,7,10,11,12]. Such an approach has been incorporated into the ITER specification tungsten, which could satisfy rolling or forging processes [13,14,15]. For applications that involve high temperature and impact loading, alternative techniques, such as severe plastic deformation (SPD), high pressing torsion (HPT), and equal-channel angular pressing (ECAP), have been suggested to improve the ductility of W [8,11]. Meanwhile, in the case of fusion reactor applications, W armors are likely to be subjected to exposure at high temperatures and thermal cycles [7]. Therefore, the surface temperature of W armors in fusion reactors is likely to reach high temperatures, exceeding the recrystallization temperature of W, leading to a change in microstructure [16,17,18]. In addition, thermal shock events would be imposed on W mono-blocks during transient operation, such as plasma disruption or edge-localized modes, which deposit high-heat loads to the divertor surface in a short duration [13,18]. In such a case, it is probable that intentionally produced dislocations during TMP would mostly disappear due to recrystallization during operation. Further, recrystallized W armor is likely to go through high-strain-rate deformation under transient operation. Previously, the mechanical behavior of commercial-grade pure tungsten in a high temperature range has been investigated [19,20]. Nonetheless, studies on the effect of recrystallization condition and strain rate on different initial microstructure evolution and associated mechanical property changes are still lacking.

This study aims to investigate the strain-rate-dependent deformation behavior of commercial-grade W depending on initial microstructure. In order to prepare recrystallized W (RX-W), double-forged Plansee W (DF-W) was heat treated at 1300 °C for 3 h. By using tensile tests under various strain-rate conditions (1.3 × 10^−^^4^–5.3 × 10^−^^1^ s^−^^1^) at 700 °C, the effect of initial microstructure and strain rate on tensile deformation behavior was evaluated. The microstructural evolution for tensile-tested samples was characterized using electron backscatter diffraction (EBSD) analysis. The dynamic microstructure evolution and associated deformation behavior were analyzed to understand the abnormal mechanical behavior of W in terms of initial microstructure and strain rate.

## 2. Materials and Methods

The tungsten material used in this study is double-forged W in the form of a cylindrical rod. A piece of DF-W was heat treated at 1300 °C for 3 h to prepare recrystallized W. The dog-bone-type tensile specimens with gage length of 15 mm and thickness of 3 mm were machined with tensile axis parallel to the longitudinal direction of the rod. The tensile tests were performed at 700 °C with various strain-rate conditions (1.3 × 10^−^^4^–5.3 × 10^−^^1^ s^−^^1^) for both DF-W and RX-W. Prior to loading tensile specimen, entire surfaces of specimen were coated by thermal spray for high-temperature oxidation protection. The tensile specimen and a loading fixture were positioned in a preheated box furnace and held for 30 min to achieve homogenization of temperature in specimen. During tests, Ar gas was continuously injected into the sample surface to minimize the effect of oxidation on tensile property. After tensile tests, microstructural evolution was carried out using electron backscatter diffraction (EBSD) analysis in the vicinity of fracture surface. The EBSD samples were machined by electrical discharge machining (EDM) and ground down to 0.05 μm colloidal silica. The cross-sectional EBSD analysis was performed using a field emission scanning electron microscope (FE-SEM, JSM-7100F, Jeol Ltd., Tokyo, Japan) using a step size of 80 nm with acceleration voltage of 15 kV. The misorientation angles were indicated in each map to identify low-angle grain boundaries (LAGBs, 2° ≤ θ < 15°) and high-angle grain boundaries (HAGBs, θ ≥ 15°). In order to investigate dynamic evolution of microstructure, post-data processing was conducted using orientation imaging microscopy (OIM) software (EDAX, TSL analysis v8, Mahwah, NJ, USA), which is capable of identifying inverse pole figure (IPF), grain orientation spread (GOS), GROD (grain reference orientation deviation), grain shape aspect ratio, and density of the geometrically necessary dislocations (GNDs).

## 3. Results and Discussion

### 3.1. Microstructure Change after Recrystallization Heat Treatment

**Figure 1:**
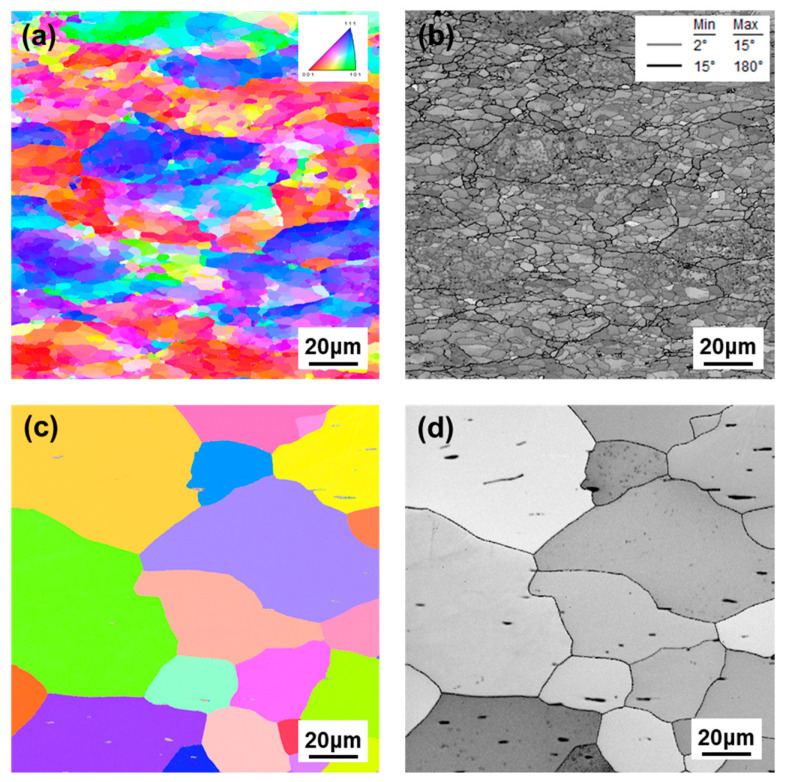
The inverse pole figure (IPF) and grain boundary misorientation maps (LAGBs (2° ≤ θ < 15°) and HAGBs (θ ≥ 15°) are indicated by gray and black lines, respectively) of undeformed W samples; (**a**) and (**b**) DF-W, (**c**) and (**d**) RX-W.

Figure 1 represents the IPF and misorientation maps obtained from EBSD for DF-W and RX-W specimens. As shown in Figure 1a,b, DF-W exhibits a typical sub-grain structure with color contrast within large grains with certain crystallographic orientation caused by the deformation during the forging process (Figure 1a). The grain boundary (GB) character measured by the grain misorientation map (Figure 1b) shows GBs mostly consist of LAGBs, nearly ~80%. On the other hand, for RX-W, most of the LAGBs disappeared and large equiaxed grains with HAGBs were present, indicating recrystallization and grain growth occurred (Figure 1c,d).

### 3.2. Strain-Rate-Dependent Tensile Behavior

**Figure 2:**
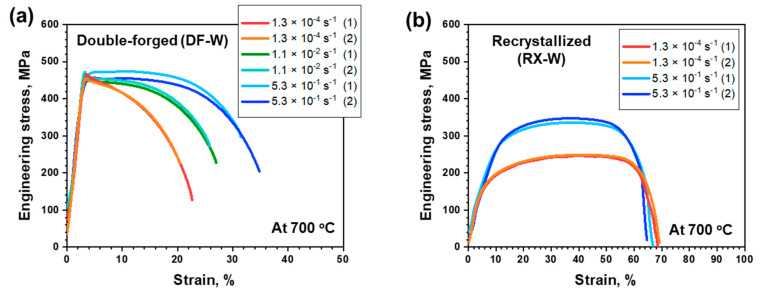
The stress–strain curves of W under various stain rate conditions: (**a**) DF-W and (**b**) RX-W.

The engineering stress–strain (S-S) curves of tensile-tested specimens under various strain-rate conditions at 700 °C for DF-W and RX-W are shown in Figure 2. Under the static-loading condition (strain rate of 1.3 × 10^−4^), DF-W shows rather high yield strength (YS), ~430 MPa, and the onset of plastic instability was subsequently observed with negligible strain hardening. Post-necking deformation was continued until 22% of strain, when final failure occurred. Meanwhile, for RX-W, strain-hardening capacity with large uniform elongation with ~70% of total elongation was observed, while YS decreased to ~110 MPa. Such behaviors could be attributed to a difference in their initial microstructure and dislocation density [21,22]. That is, high dislocation density that existed in DF-W could result in a dislocation–dislocation interaction, which interferes with their movements and results in high YS and reduced ductility. On the other hand, for RX-W, the absence of pre-existing dislocations could lower critical stress to induce dislocation movement and increase the capability of dislocation accumulation.

Changes in stress–strain curves with increasing strain rate follow different pathways depending on the initial microstructure. For DF-W, there is no noticeable change in yield strength despite an increase in strain rate by more than 3 orders of magnitude (Figure 2a). However, post-necking strain to final fracture was gradually increased with the strain rate while flow stress beyond necking shows flow-softening behavior, regardless of strain rate. The continuous decrease in flow stress is caused by dynamic softening, which suggests that restoration is more dominant than work hardening related to dislocation generation/interaction [23,24,25]. Meanwhile, after necking emerges, flow stress under a high-strain-rate condition is higher than that in the static-loading condition, indicating DF-W under a higher strain rate undergoes a relatively weaker restoration process. On the other hand, in the case of RX-W, an increase in YS with a slight reduction in ductility was clearly observed, while strong strain-hardening capability was maintained in the high-strain-rate condition (Figure 2b). It could be said that work hardening still dominates plastic deformation rather than dislocation restoration due to the low density of existing dislocation.

### 3.3. Effect of Initial Microstructure and Strain Rate on Dynamic Recrystallization Behavior

**Figure 3:**
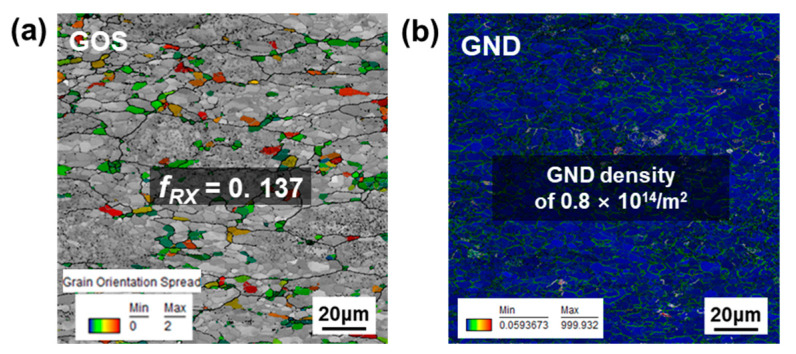
The EBSD analysis results of undeformed DF-W sample: (**a**) GOS map and (**b**) GND map.

**Figure 4:**
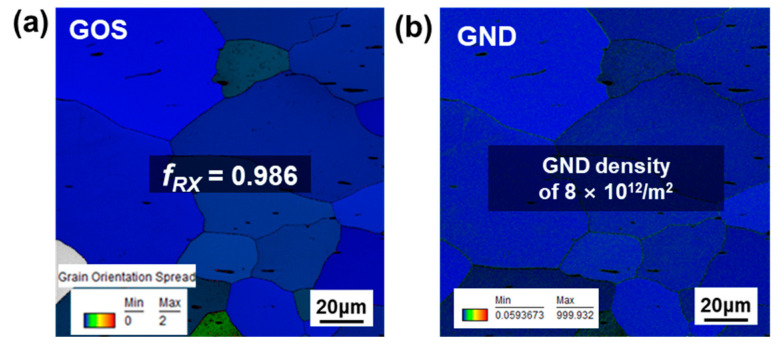
The EBSD analysis results of undeformed RX-W sample: (**a**) GOS map and (**b**) GND map.

**Figure 5:**
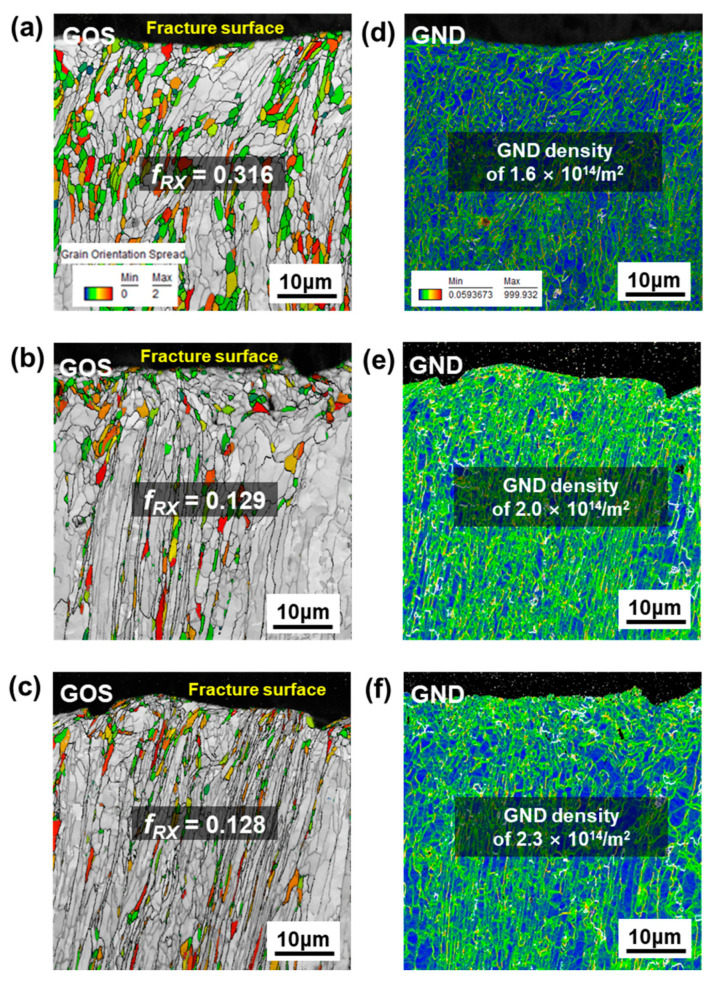
The GOS and GND maps of DF-W sample after tensile tests: (**a**,**d**) static (1.3 × 10^−4^ s^−1^), (**b**,**e**) intermediate-strain-rate (1.1 × 10^−2^ s^−1^), (**c**,**f**) high-strain-rate (5.3 × 10^−1^ s^−1^) conditions.

**Figure 6:**
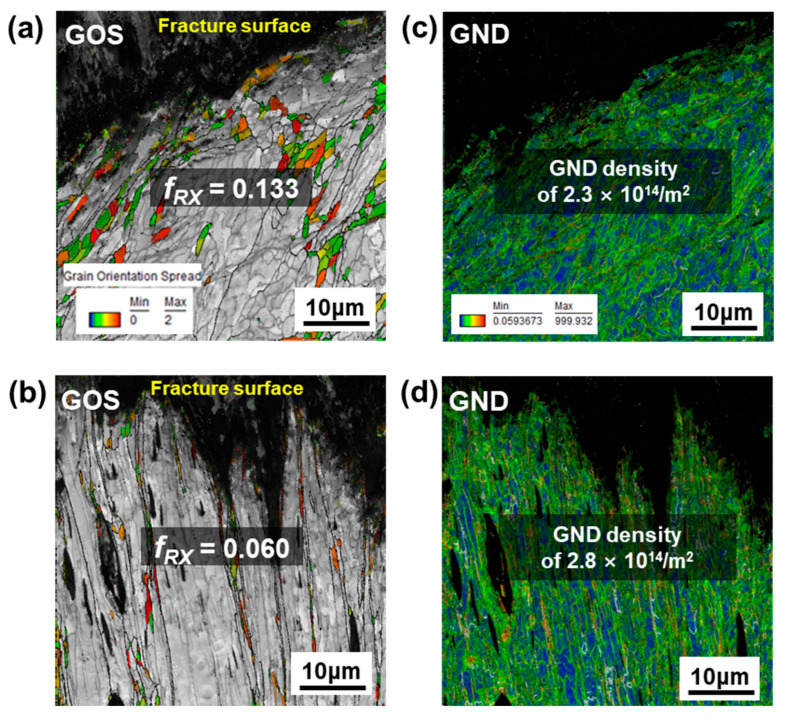
The GOS and GND maps of RX-W sample after tensile tests: (**a**,**c**) static (1.3 × 10^−4^ s^−1^), (**b**,**d**) high-strain-rate (5.3 × 10^−1^ s^−1^) conditions.

In order to characterize the microstructural features responsible for strain-rate-sensitive deformation behavior, GOS and GND analyses were performed in the vicinity of fracture surface and the results are shown in Figure 3, Figure 4, Figure 5 and Figure 6. The definition of GOS is the average deviation in orientation between each point in a grain and the average orientation of the grain [23]. Therefore, the deformed grains with a number of dislocations have high GOS values, while recrystallized grains free of dislocations have relatively lower GOS values. According to previous studies, it was reported that GOS could be used to identify DRX grains, such that particular grains with a GOS value less than 2° could be considered as DRX grains [23,26]. GND could be analyzed on the basis of Nye’s concept from local misorientation angle between neighboring lattices [5]. It was reported that the entire dislocation density derived from the XRD profile analysis using the broadening of diffraction peak shows a similar tendency of the GND analysis results from EBSD for commercial-grade pure tungsten [5].

Figure 3 represents GOS and GND maps for undeformed DF-W sample. The recrystallized grains whose GOS values were less than 2° are indicated by color (the region in gray which has GOS > 2° represents deformed grain). In the case of undeformed DF-W in Figure 3a, the GOS of grains mostly shows greater than 2°, suggesting dislocation accumulation took place during the forging process. Further, well-developed sub-grain structures signify the occurrence of DRV during the fabrication process. However, for the RX-W condition, all the grains in Figure 4a show lower GOS value than 2°, indicating low dislocation density in the undeformed sample. The area fraction of recrystallized grains, denoted by *f_RX_*, measured by the GOS map, corresponds to 0.986. The estimated GND density of RX-W shown in Figure 4b is nearly 8 × 10^12^/m^2^, which is the same order of magnitude as that in common recrystallized W [5]. After the forging process, GND density increases to 0.8 × 10^14^/m^2^ while relative *f_RX_* decreases to 0.137 (Figure 3a,b).

After tensile deformation of the DF-W sample, *f_RX_* measured in GOS maps shows a decreasing tendency with increasing strain rate in Figure 5. Under the static-loading condition, well-developed sub-grains and DRX grains (GOS ≤ 2°) were rather uniformly distributed with a higher value of *f_RX_*, nearly ~0.32, higher than that of the undeformed condition (Figure 5a). Meanwhile, at higher strain rates, somewhat different deformation morphology was present with the combination of elongated grains and fewer recrystallized grains in the vicinity of elongated grains rather than the formation of sub-grain structure (Figure 5b,c). The *f_RX_* (approximately 0.13) was nearly 2.5-times lower than that in the static-loading condition, which suggests a predominant restoration mechanism might be altered with increasing stain rate.

For the RX-W condition, a similar microstructural pathway was observed, as shown in Figure 6a,b. In the case of the deformed sample under the static-loading condition, a well-developed sub-grain structure is clearly visible and equiaxed DRX grains are present in the entire sample homogeneously, though *f_RX_* (~0.13) is significantly less than that of DF-W (Figure 5a). With increasing strain rate up to 5.3 × 10^−1^ s^−1^, deformed grains stretching along tensile loading axis were visualized with DRX grains in the vicinity of elongated grains with relatively lower *f_RX_* (~0.06) in Figure 6b. It is likely that the increase in strain rate would result in a shift in the DRX mechanism and suppression of *f_RX_*, the tendency of which is consistent with that in DF-W.

Previously, it was reported that a different type of DRX was observed in pure Mo [27], Mo-Ti-Zr (Mo-MZM) [23,28], and various metallic alloys [24,25] during hot deformation. When new grains free of strain are nucleated in a discontinuous manner at the cost of strained grain, this process is categorized as discontinuous DRX (DDRX). In some cases, sub-grain structure with LAGB is developed at lower strain via DRV and they would evolve into HAGB through progressive cumulative misorientation at further deformation [23,24,25,27,28]. This process corresponds to continuous DRX (CDRX). In this case, the microstructure evolution caused by CDRX shows a somewhat homogeneous aspect without distinguishable nucleation and growth of the newly formed grain [28]. Furthermore, CDRX could be accelerated under a lower-strain-rate condition because of enough time for dislocations to climb or slip into sub-grain boundaries [23,25,27,28]. According to previous studies, an increase in strain rate may have a similar effect in decreasing the temperature or γ_SF_ [29]. Therefore, higher-strain-rate deformation could prevent the formation of dislocation cells or sub-grains and alter overall slip character from wavy to planar slip in several metals [28,29].

**Figure 7:**
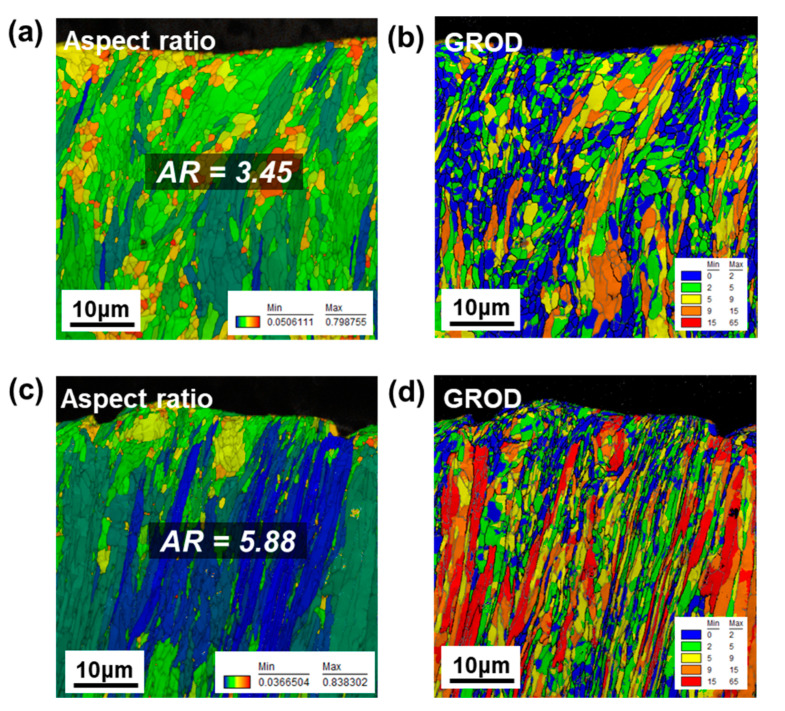
The grain aspect ratio and GROD maps of deformed DF-W sample: (**a**,**b**) static (1.3 × 10^−4^ s^−1^); (**c**,**d**) high-strain-rate (5.3 × 10^−1^ s^−1^) conditions.

**Figure 8:**
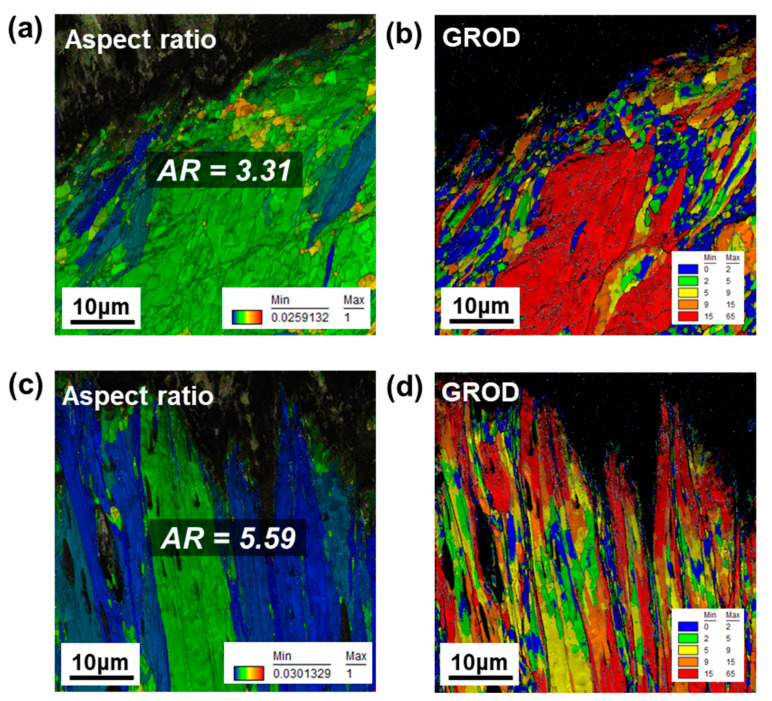
The grain aspect ratio and GROD maps of deformed RX-W sample: (**a**,**b**) static (1.3 × 10^−4^ s^−1^); (**c**,**d**) high-strain-rate (5.3 × 10^−1^ s^−1^) conditions.

The signatures of DDRX are elongated grains and small fraction of DRXed grains in the vicinity of the boundaries of elongated grains (necklace nucleation), because DDRX is related to the grain boundary shear stress that causes inhomogeneous strain as the driving force of DDRX [23,27,28,29]. As shown in Figure 7a,c, the aspect ratio of deformed grains shows an increasing tendency for the DF-W condition with increasing strain rate, implying a shift in recrystallization behavior with increasing strain rate. It was also reported that the high aspect ratio of grain shape in the deformed sample is positively correlated with the frequency of DDRX [30]. Furthermore, under the DDRX process, the grains are elongated with the high-strain accumulation due to insufficient time for any dynamic recovery process [23,30]. The GROD (grain reference orientation deviation) maps that represent the distribution of stored energy depending on strain rates are illustrated in Figure 7b,d. The highest value of GROD at the lower-strain rate (1.3 × 10^−4^ s^−1^) is lower than that in at high-strain rate (5.3 × 10^−1^ s^−1^), indicating the occurrence of dynamic recovery and CDRX under the lower-strain rate reduced the strain accumulation (DRXed grain depicted as blue in Figure 7b) [23]. Further, for deformation at a low-strain rate, the distribution of strain and potent sites for DRX grains seems to be homogeneous. Meanwhile, for the high-strain-rate condition, high-strain accumulation inside grains (depicted as red color in Figure 7d) was non-uniformly observed and the reduced DRX was detected usually in the vicinity of the high-strain-accumulated region, colored as orange or red. The heterogeneous microstructure is a signature of partial DRX, suggesting a shift in the DRX mechanism is responsible for the reduced fraction of DRX [23,27]. Even for the RX-W condition, similar DRX behavior was observed, as shown in Figure 8, in terms of aspect ratio and strain accumulation. Therefore, it could be said that the main dynamic softening mechanism for both DF-W and RX-W could be shifted from CDRX to DDRX with increasing strain rate, leading to lower *f_RX_*.

### 3.4. Effect of Initial Microstructure and Strain Rate on GND Evolution

In order to investigate the strain localization of deformed W, GND maps of DF-W were constructed in Figure 5d–f. For the static-loading condition with high *f_RX_*, GNDs seem to be relatively homogeneously distributed in the entire area with lower strain accumulation within grains, suggesting the occurrence of CDRX reduces GNDs inside grains (Figure 5a,d). With an increasing strain rate, high strain was mostly imparted into elongated grain (deformation band) and a small fraction of equiaxed recrystallized grains was decorated near pre-existing grain-boundaries by DDRX (Figure 5e,f). Therefore, a relatively high density of residual dislocations at higher strain rate in GND maps indicates a weaker restoration process occurred due to a shift in DRX mechanism from CDRX to DDRX (Figure 5d–f).

In the case of RX-W, the evolution of GNDs with increasing strain rate shows a similar tendency when compared to that in DF-W through a similar DRX mechanism (Figure 6c,d). Regardless of initial microstructure, as mentioned earlier, an increase in strain could inhibit the formation of a dislocation cell or sub-grains, though cross-slip could be easily achieved due to its inherent high γ_SF_ [27,29]. Further, it was reported that deformed microstructure under higher strain rate is likely to be long and straight dislocations rather than tangling or dislocation cells in niobium [29]. According to results of GND density (Figure 5 and Figure 6) and GROD (Figure 7 and Figure 8) analyses, for both DF-W and RX-W, the high strain accumulation inside grains could be caused by a change in predominant dislocation substructure with increasing strain rate. Nonetheless, measured GNDs in RX-W represent higher value than that of DF-W for all the strain-rate conditions (Figure 5 and Figure 6), which could be attributed to a difference in pre-existing dislocation density in undeformed samples.

It is worth noting that flow behavior seems to be totally different, although strain-rate-dependent microstructure change and governing restoration mechanism are likely to be identical for both DF-W and RX-W conditions. For the DF-W condition, in the case of the static-loading condition, pre-developed sub-grain structure with higher density of existing dislocations may suppress strain hardening and promote the CDRX process with increasing strain due to pre-existing sub-grains, resulting in accelerating flow softening. However, a shift in the DRX mechanism to DDRX under a high-strain rate could result in higher dislocation density and associated high flow stress that lead to an increase in strength in the diffuse necking region, contributing to ductility enhancement during the post-necking process [31]. On the contrary, in the case of RX-W, due to initially lower dislocation density and large grain size, lower YS and higher strain-hardening behavior were prominent, indicating dislocation accumulation surpassed annihilation of dislocation, regardless of strain rate. Increasing strain rate would lead to the suppression of DRX and a resultant dislocation density increase, leading to slightly reduced total elongation and flow hardening.

## 4. Conclusions

The tensile deformation behavior for DF-W and RX-W was investigated under various strain rates of 1.3 × 10^−4^–5.3 × 10^−1^ s^−1^ at 700 °C. Under the static-loading condition, DF-W exhibits high yield strength and ~22% of total elongation, but with negligible work hardening. After recrystallization at 1300 °C for 3 h, RX-W shows significant work hardening with enough ductility, ~70%. With increasing strain rate, DF-W represents enhanced ductility with a negligible change in YS, while RX-W undergoes significant hardening in YS and a slight reduction in ductility. The deformation morphology analyzed by EBSD suggests that CDRX is the main restoration mechanism in the static-loading condition, but high-strain-rate-condition DDRX played a major role in dynamic softening behavior for both materials. Meanwhile, in the case of DF-W, pre-existing sub-grained structure seems to promote CDRX under the static-loading condition, which leads to high-DRX fractions and associated flow softening. The shift in restoration mechanism from CDRX to DDRX with increasing strain rate could be responsible for the enhanced ductility of DF-W, because of strengthening in the necking region. On the other hand, RX-W shows a different pathway in flow behavior. Increasing strain rate leads to an increase in dislocation density from lower DRX fraction, which results in flow hardening with slightly reduced ductility.

## Data Availability

The raw/processed data required to reproduce these findings cannot be shared at this time due to technical or time limitations. They will be shared upon request.
